# Effects of Website Interactivity on Skin Cancer–Related Intentions and User Experience: Factorial Randomized Experiment

**DOI:** 10.2196/18299

**Published:** 2021-01-13

**Authors:** Zhaomeng Niu, Jessica Fitts Willoughby, Elliot J Coups, Jerod L Stapleton

**Affiliations:** 1 Section of Behavioral Sciences Division of Medical Oncology Rutgers Institute of New Jersey New Brunswick, NJ United States; 2 The Edward R Murrow College of Communication Washington State University Pullman, WA United States; 3 Medical Data Analytics Parsippany, NJ United States; 4 Department of Health, Behavior & Society College of Public Health University of Kentucky Lexington, KY United States

**Keywords:** behavioral intention, computer-mediated communication, skin cancer, interactivity, user experience

## Abstract

**Background:**

Digital media technologies provide users with the ability to interact with content and to receive information based on their preferences and engagement.

**Objective:**

We used skin cancer and sun protection as a health topic to explore how modality interactivity, interface tools that afford users greater activity, resulting in greater depth and breadth of mentally representing and experiencing mediated content, and message interactivity, the extent to which the system allows users to exchange messages back and forth on health websites, influenced users’ attitudes, knowledge, behavioral intentions, and experience.

**Methods:**

We employed a 2×2 (modality interactivity: high vs low; message interactivity: high vs low) between-subject online experiment for which 4 websites were created. Participants (n=293) were recruited using Amazon Mechanical Turk and randomly assigned into to 1 of 4 conditions. After browsing the website, participants completed an online survey regarding their experience and cognitive perceptions. General linear models and path analysis were used to analyze the data.

**Results:**

Both modality interactivity (*P*=.001) and message interactivity (*P*<.001) had an impact on intention to use sun protection. Attitudes toward health websites and perceived knowledge mediated the effects of modality interactivity and message interactivity on sun protection use intention, individually. Participants in the high modality interactivity and high message interactivity condition felt more satisfied (*P*=.02). Participants in the low message interactivity condition had more interest in the experience with health websites than participants in the high message interactivity condition (*P*=.044).

**Conclusions:**

Findings suggested that modality interactivity influenced intention to use sun protection directly as well as via attitudes toward the websites. Message interactivity impacted intention to use sunscreen directly and also through perceived knowledge. Implications for designing health websites and health intervention content are discussed.

## Introduction

### Background

The use of technology in communication is ubiquitous in today’s society. As a result, communication has shifted from traditional 1-way communication to 2-way reciprocal approaches [1,2]. Media users become active information seekers instead of passive information receivers. The integration of advanced elements of interpersonal communication and mass communication into internet-based communication has resulted in immediate, back-and-forth, customized responses [3,4]. Moreover, users can generate content and share experiences with other users, and users themselves may become sources as well as receivers of information [5,6]. This 2-way flow of communication has been useful for health promotion efforts [7-13].

According to the Pew Internet & American Life Project [14], 1 in 3 Americans have used the internet to solve a medical problem. Due to the public’s increasing willingness to be more responsible for their own health [15,16], how to effectively deliver health information using technologies has become a question requiring further investigation. Internet-based interventions were found to work better than noninternet-based interventions for improving a variety of health-related attitudes and behavioral outcomes including increased knowledge, participation in health care, and more [17].

Interactivity, defined as how responsive a system is to a user [18], has been examined by many researchers in health communication as a key characteristic of interactive digital media technologies [19-24]. While some have found that higher levels of interactivity can improve knowledge [20], attitude toward health websites [21,24], and intentions to use a health information resource [25,26], others have found that higher levels of interactivity did not have positive effects on knowledge [22,27] or self-efficacy [22]. Overloading of technological features on media interfaces (exceeding the cognitive processing boundary) may lead to distraction and reactance to the persuasive messages [28-30]. Since there are both arguments for and against using interactive technological affordances, in terms of information processing and assessment, it is important to further examine the role of interactive features in an online environment.

We aimed to examine (1) how different types of interactivity influence health behavioral intentions and whether there is an interaction effect among these 2 types of interactivity on behavioral intentions; (2) how different types of interactivity impact behavioral intentions through different cognition; (3) how different types of interactivity influence individual user experience with health websites.

### Skin Cancer and Sun Protection

Skin cancer is the most common cancer in the United States [31]. One in 5 Americans will develop skin cancer in their lifetime [32]. Each year, more than 3.5 million new cases of skin cancer are diagnosed in the United States, which is more than the yearly total of new cases for all other types of cancers [33,34]. However, skin cancer is the most preventable cancer compared to other forms of cancer [35]. The most preventable risk factor is exposure to ultraviolet light [35]. Therefore, most skin cancer interventions aim to promote sun protection behaviors, such as using protective clothes or shades and increasing sunscreen use in the general public or in people at high risk of skin cancer or decreasing exposure to artificial ultraviolet light (eg, tanning beds). Researchers have been working on building effective skin cancer interventions for decades [36-43]; however, no previous study has employed skin cancer as a health context to examine how different types of interactivity affect user experience with health websites, behavioral intentions, and information processing [21].

### Interactivity

According to the Modality, Agency, Interactivity, and Navigability (MAIN) model [6], interactivity should be a system of technological affordances that can allow users to make changes to sources, messages, and media while interacting with the interfaces. Based on the above, there are 3 types of interactivity—modality interactivity, message interactivity, and source interactivity—and they can influence user engagement then sequentially affect cognition, attitude, and behavior [6,44].

When interactivity is assessed as a functional modality on the medium interface, it is called *modality interactivity*. Modality interactivity refers to “interface tools that afford users greater activity, resulting in greater depth and breadth of mentally representing and experiencing mediated content [44].” Traditional media usually contain a single modality. For example, print media, may solely have text, and radio may solely provide audio. Digital media have multiple modalities as multimedia content [45,46]. Modality cues not only include video, image, text, and audio but also include new interactive affordances such as hyperlinks, clicking, zooming, dragging, scrolling, and mouse-overs.

Message interactivity has been demonstrated by many researchers through the concept of message exchange [18] or 2-way communication [47]. *Message interactivity* refers to the extent to which the system allows users to exchange messages back and forth. The action of message interactivity is performed through the principle of contingency, which means that “the idea that a given message is contingent upon user reception of the previous message and the ones preceding that [44].” If a system or a media channel allows users to have back and forth interaction in a highly logical flow, the system or the channel is seen as having high message interactivity. Hyperlinks and buttons embedded in websites are a typical format for manipulating message interactivity. Sundar et al [48] examined the effect of how hypertext, when operationalized as message interactivity, allowed users to access information through nonlinear communication while exploring a website.

Previous studies [49-52] have found that modality interactivity can influence cognitive perceptions including attitudes and behavioral intentions. Many empirical studies [20,24,53] have shown the effectiveness of modality interactivity in health communication, such as increasing attitudes toward health websites and physical activity intentions. However, few studies have specifically investigated message interactivity on attitudes and intentions under the context of health. One study [44] found that higher message interactivity can lead to higher evaluation of the content and result in higher intentions to recommend the website to others.

### Research Questions and Hypotheses

Based on previously reviewed literature, prior research mainly focused on the effectiveness of interactivity and compare the effects of different levels of interactivity; little research has been conducted to examine the interaction effects of different types of interactivity and whether there is an interaction effect between different types of interactivity. However, in real-life situations, different types of interactivity usually are presented together on the interface, and individuals interact with multiple technological affordances back and forth, curvilinearly. Furthermore, there has been limited research investigating how different types of interactivity influence user experience factors, such as satisfaction and interest.

Thus, we used skin cancer as the context and proposed the following hypotheses and research questions—

Hypothesis 1: Modality interactivity will be positively associated with attitudes toward a health website and intention to use sun protection.

Hypothesis 2: Message interactivity will be positively associated with knowledge of skin cancer and intention to use sun protection.

Research question 1: Is there an interaction effect on attitudes, knowledge or intention?

Hypothesis 3: Modality interactivity will mediate behavioral intention via its prior effects on attitudes toward health websites.

Hypothesis 4: Message interactivity will mediate behavioral intention via its prior effects on knowledge.

Research question 2: How will modality interactivity and message interactivity impact user satisfaction of and user interest in the experience with health websites?

## Methods

### Design Overview

This study was a 2×2 (modality interactivity: high vs low; message interactivity: high vs low) online experiment to evaluate effects of modality interactivity and message interactivity on user experience, knowledge, attitudes, and behavioral intentions.

### Participants and Sample

Participants were recruited from Amazon Mechanical Turk, an online crowdsourcing marketplace for tasks, which are posted on the platform. The platform can provide a more diverse and valid sample according to previous studies [54,55]. Participants who had a preexisting account on Mechanical Turk took part in the study. Two attention checks were added in the survey. One is the commonly used instructional manipulation checks, which can increase the statistical power and reliability of a data set [56]. In this attention check, participants must not click on anything and directly go to the next page. The other attention check was a statement at the end of a set of questions asking the participants to choose one specific number to make sure they were reading the questions. Data from individuals who did not pass these 2 checks were not included in the data set. We also had manipulation checks for each type of interactivity. For the modality interactivity, participants were asked if they dragged the slider bar to view the change of the pictures. If the respondents answered “no” to the question, they were dropped from the data. For message interactivity, participants were asked if they clicked on the plus icon to view more information about sun protection. The respondents who answered “no” were dropped as well. Additionally, perceived interactivity was used to evaluate the interactivity level in each condition.

### Procedure

An online questionnaire (Qualtrics XM) was used to collect data from the respondents. All the independent variables were between-subject factors. Participants gave consent to participate in this study and were randomly assigned to 1 of 4 experimental conditions. Each participant was provided with a link and asked to explore the websites as much as possible. They were instructed to read all the information on the website and click on as many links and buttons as possible. After browsing the website, each participant completed a questionnaire regarding the website, their perceptions about skin cancer, and sun protection and provided demographic information. At the beginning of the survey, a prompt asking them if they had browsed the website. Such reminders have been found to be one way to increase the viewing of content. The incentive for each participant was US $0.50. The study was approved by Washington State University's institutional review board.

### Experimental Treatment Conditions

**Figure 1:**
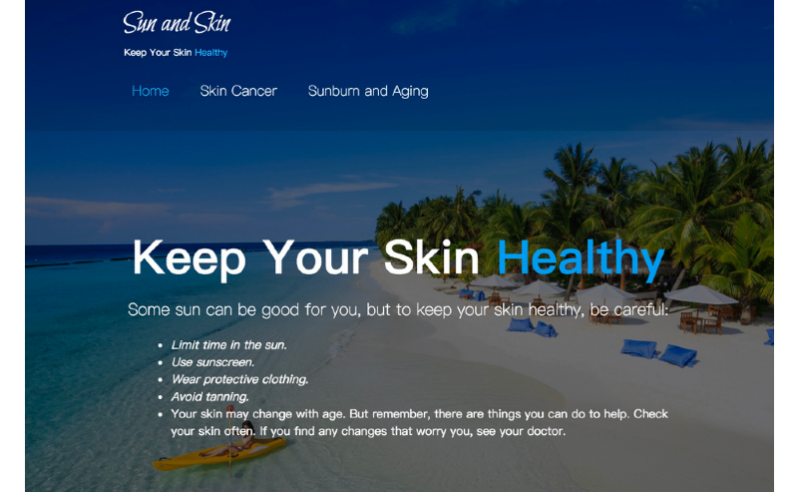
Screenshot of the website homepage.

Four websites were built for this research project. All 4 websites had the same webpage layout and health content. They only differed in terms of interactive features. The websites’ title was “Sun and Skin” with sections on the webpage including one about skin cancer and the other one about sunburn and aging (Figure 1).

**Figure 2:**
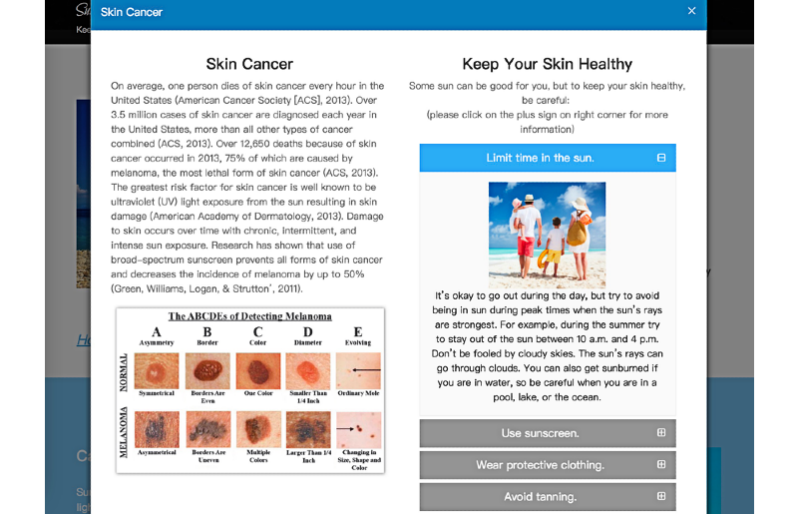
Example of the high message interactivity webpage.

Message interactivity was manipulated in the Skin Cancer section of the websites. High message interactivity condition had a clicking function, which the participants needed to click on the bars to get further information (Figure 2). The low message interactivity condition did not have the clicking function, and participants could read the information by scrolling down the page. This is consistent with previous manipulations of message interactivity [24].

**Figure 3:**
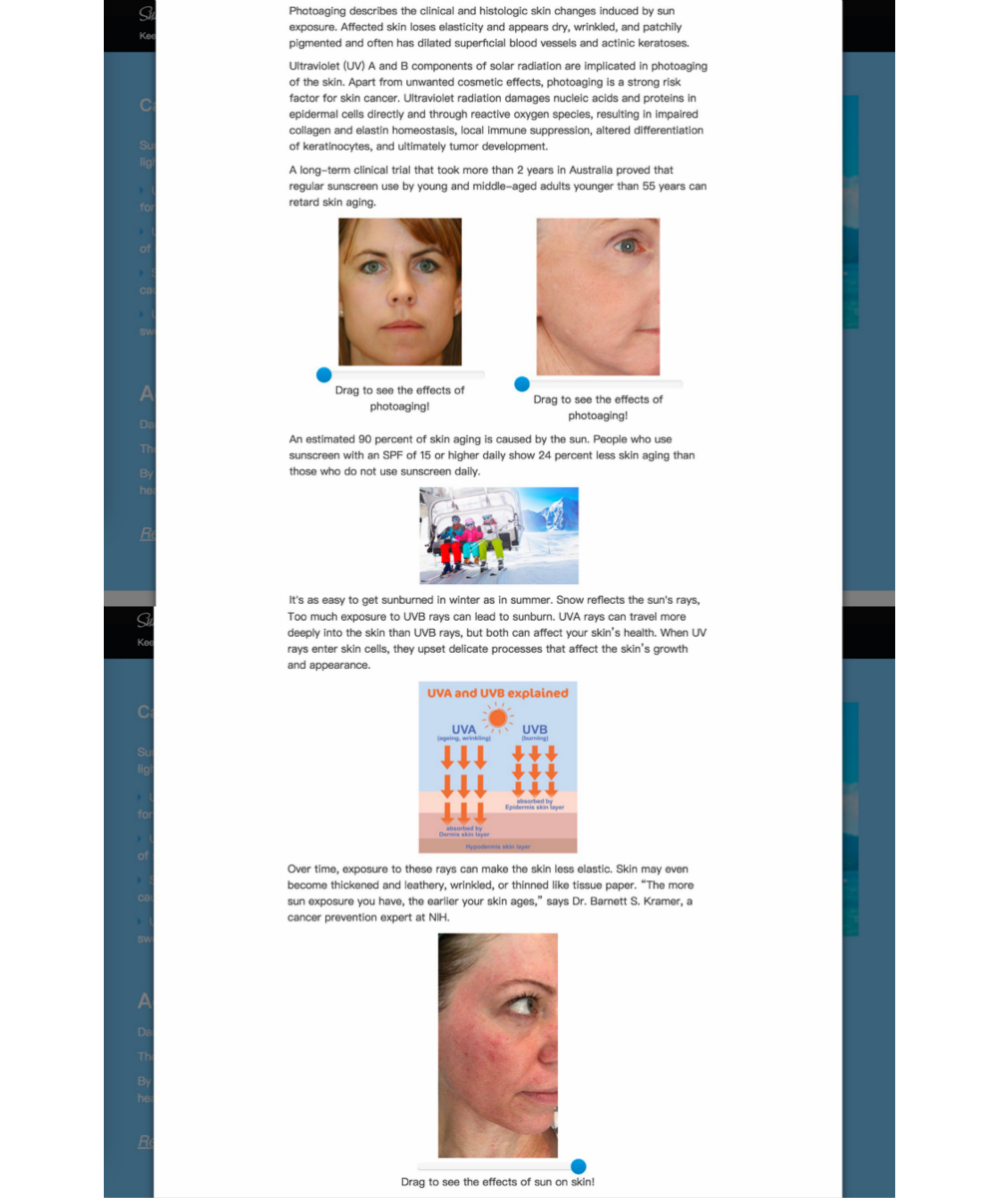
Example of high modality interactivity webpage.

Modality interactivity was manipulated in the Sunburn and Aging section of the websites. The high modality interactivity condition had a slider bar feature, which the participants could slide from left to right to view the process of aging (Figure 3). In the low modality interactivity condition, 2 pictures of aging were directly placed on the webpage. This is consistent with previous manipulations of modality interactivity [24].

### Measures

#### Manipulation Checks

Perceived interactivity was measured by 3 items adapted from Kalyanaraman and Sundar [5], asking participants to indicate how interactive the website was, if the website allowed them to perform a lot of actions, and if the website allowed them to access information in a variety of ways. Additionally, a manipulation check item was asked for each of the interactivity manipulation types to assess exposure to the stimuli. To assess message interactivity, participants were asked “Did you click on the link to view more information?” (yes or no response) and “How interactive did you feel the site was when using the drop-down button/menu?” (scale of 1-7, where 1=not at all and 7=extremely). Modality interactivity was assessed with “Did you drag the slider bar to view more information?” (yes or no response) and “How interactive did you feel the site was while dragging the slider to view the picture?” (scale of 1-7, where 1=not at all and 7=extremely).

#### Dependent Variables

Behavioral intention to use sunscreen was measured by 3 items (using a 7-point Likert scale) items adapted from Kahlor [57] including “I plan to use sunscreen in the future,” “I will try to use sunscreen in the future,” and “I intend to use sunscreen in the near future” (Cronbach α=.93).

User satisfaction of the experience was measured 5 items (using a 7-point Likert scale) such as “I am totally satisfied with my interaction with this health website” and “This site was very careful in considering my health information needs” (Cronbach α=.76).

User interest in the experience was measured by 3 items (using a 7- point Likert scale) such as “The interaction with this health website was interesting” and “I am interested in the health information presented on the website” (Cronbach α=.76).

#### Mediating Variables

Attitudes toward the health websites were measured by statements asking the respondents on a 7-point scale whether they felt that the website they just viewed was exciting or not exciting, high quality or low quality, fun or not fun, cool or not cool, imaginative or not imaginative, and entertaining or not entertaining (Cronbach α=.87).

Knowledge was measured from 2 aspects, the objective memory recall and the perception of knowledge increase. Perceived knowledge was measured by 4 items (using a 7-point Likert-scale ranging from 1=strongly disagree to 7=strongly agree) asking how knowledgeable the respondents felt after exploring the websites [20], such as “I feel very knowledgeable about skin cancer” and “I feel very confident about my ability to tell the disadvantages of sun exposure” (Cronbach α=.89). Objective memory recall was measured by 3 questions asking whether they thought the following statements were correct based on what they had read. The statements were retrieved from the information on the website and included “People don't need to use sunscreen in winter,” “Tanning pills can help protect skin when you use tanning beds,” and “Sunscreen with an SPF of 10 is enough for people doing outdoors activities.” Correct answers were coded as 1 and incorrect answers were coded as 0. The number of correct answers was integrated as the final scores for the objective memory recall.

### Statistical Analysis

General linear model analyses were used to test the effects of the 2 independent variables (modality interactivity and message interactivity) on the dependent variables. To test the mediating relationships, PROCESS macro (version 3.3) was conducted using SPSS (version 25.0; IBM Corp). Age, gender, race (White vs non-White), and education were controlled as covariates in all analyses. *P*<.05 was used to determine the statistical significance level. Prior to the analysis of the data, all manipulation check items were assessed. Data from participants who did not pass were excluded from analysis. Independent sample *t* tests (2-tailed) were used to check if participants in high interactivity conditions had higher scores on the second set of manipulation check questions.

## Results

### Manipulation Checks

Participants in the high modality condition (mean 4.67, SD 1.55) scored higher on perceived interactivity than participants in the low modality condition (mean 4.22, SD 1.50; t_291_=2.58, *P=*.02); participants in the high message condition (mean 4.60, SD 1.48) scored higher on perceived interactivity than participants in low message condition (mean 4.30, SD 1.60; t_291_=1.18, *P=*.03).

### User Statistics

Of 316 participants who responded to the survey, data from 293 participants were included in the analysis (participants who were missing data on variables under study, n=7; participants who did not pass manipulation checks, n=16). Demographic characteristics of the sample are shown in Table 1.

**Table 1:** Demographics of sample.

Variable		Value
Age (years), mean (SD)		35.97 (12.02)
**Gender, n (%)**		
	Male	133 (44.6)
	Female	160 (53.7)
**Race/ethnicity, n (%)**		
	Caucasian/White	217 (74.1)
	African American/Black	28 (9.5)
	Hispanic or Latino	15 (5.1)
	Asian/Pacific Islander	29 (9.9)
	Other	4 (1.4)
**Family income in last year (US $), n (%)**		
	≤$20,000	35 (11.9)
	$20,001-$50,000	111 (37.9)
	$50,001-$70,000	52 (17.7)
	$70,001-$100,000	61 (20.8)
	$100,001-$150,000	23 (7.8)
	≥$150,000	11 (3.8)
**Education, n (%)**		
	High school degree or less	24 (8.2)
	Some college	87 (29.7)
	College degree	120 (41.0)
	Some graduate school	17 (5.8)
	Graduate degree	45 (15.4)

### Modality Interactivity

The main effect of modality interactivity on attitudes toward health websites was significant (*F*_1,283_=4.02, *P=*.045, η^2^=.014). Participants in the high modality interactivity condition (mean 4.64, SE 0.12) scored higher on attitudes toward health websites than participants in the low modality condition (mean 4.30, SE 0.12). Similarly, the main effect of modality interactivity on the intention to use sunscreen was also significant (*F*_1,283_=10.59, *P=*.001, η^2^=.036). Participants who experienced high modality websites (mean 5.70, SE 0.12) scored higher on intention to use sunscreen than participants who explored the low modality websites (mean 5.17, SE 0.12). Thus, hypothesis 1 was supported.

### Message Interactivity

The main effect of message interactivity on perceived knowledge was significant (*F*_1,283_=12.08, *P=*.001, η^2^=.041). Participants in the high message interactivity condition (mean 4.98, SE 0.10) scored higher on perceived knowledge than participants in the low message condition (mean 4.47, SE 0.11). However, the main effect of message interactivity on objective memory recall was not significant. Thus, the first part of hypothesis 2 was partially supported.

The main effect of message interactivity on intention to use sunscreen was significant (*F*_1,283_=17.02, *P<*.001, η^2^=.057). However, participants in the low message interactivity condition (mean 5.78, SE 0.12) reported higher intention to use sun protection than participants in high message interactivity condition (mean 1.10, SE 0.12). Thus, the second part of hypothesis 2 was supported.

### Interaction Effects

No interaction effect was found for attitudes toward health website, perceived knowledge, or intention to use sunscreen. However, the interaction effect of modality interactivity and message interactivity on objective memory recall was significant (*F*_1,283_=12.90, *P<*.001, η^2^=.043), answering the first research question. Participants in high modality and high message interactivity group (mean 0.61, SE 0.08) scored higher on objective memory recall than participants in high modality and low message interactivity group (mean 0.20, SE 0.08).

### Mediation Analysis

**Figure 4:**
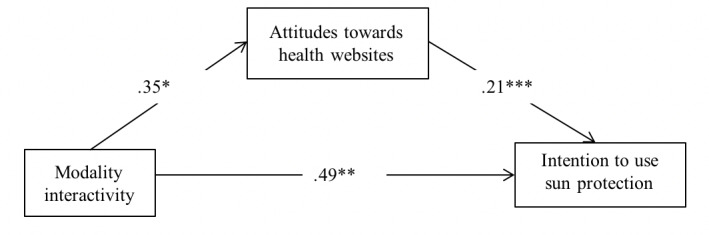
Mediation model of modality interactivity on intention through attitudes, including effects of control variables (age, sex, race, and education, which are not displayed). *P<.05; **P<.01; ***P<.001.

According to the results of mediation analyses, there was a significant indirect effect of modality interactivity on intention to use sun protection via its prior effect on attitudes toward health websites (*B*_indirect_=0.072, SE.044, 95% CI 0.003 to 0.173) (Figure 4). Thus, hypothesis 3 was supported.

**Figure 5:**
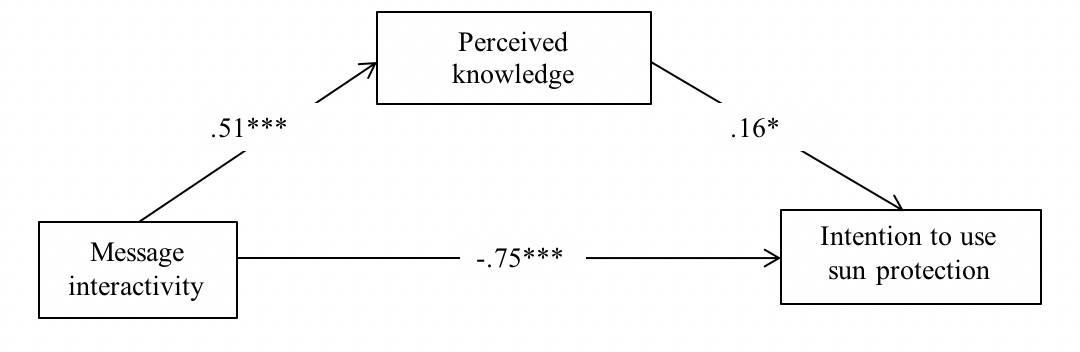
Mediation model of message interactivity on intention through attitudes, including effects of control variables (age, sex, race, and education, which are not displayed). *P<.05; **P<.01; ***P<.001.

In addition, there was a significant indirect effect of message interactivity on intention to use sun protection via its prior effect on perceived knowledge (*B*_indirect_=0.082, SE 0.046, 95% CI 0.009 to 0.187) (Figure 5). However, objective knowledge gain was not a mediator of message interactivity on intention to use sun protection. Thus, hypothesis 4 was partially supported.

### User Satisfaction and User Interest

**Figure 6:**
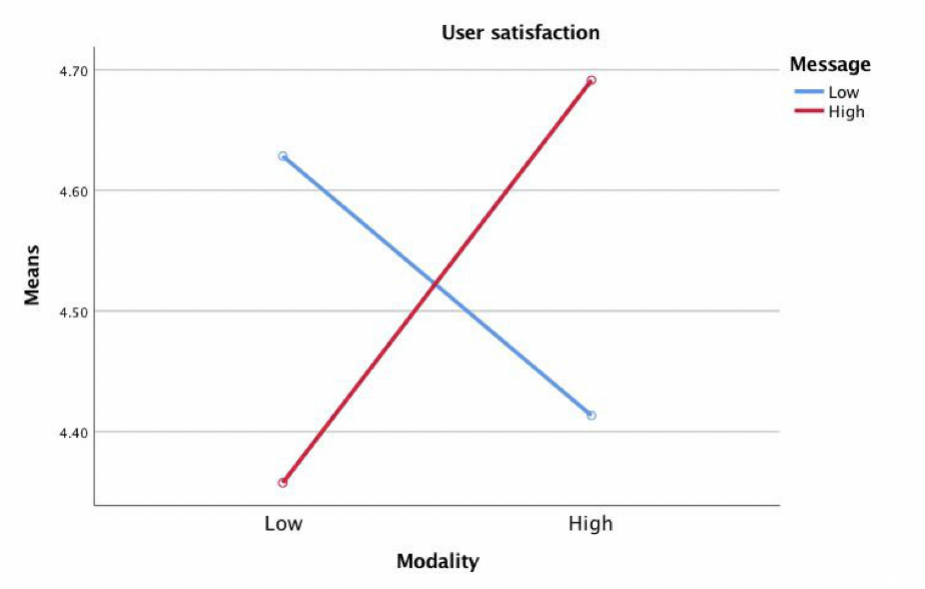
Modality interactivity and message interactivity effect on user satisfaction.

**Figure 7:**
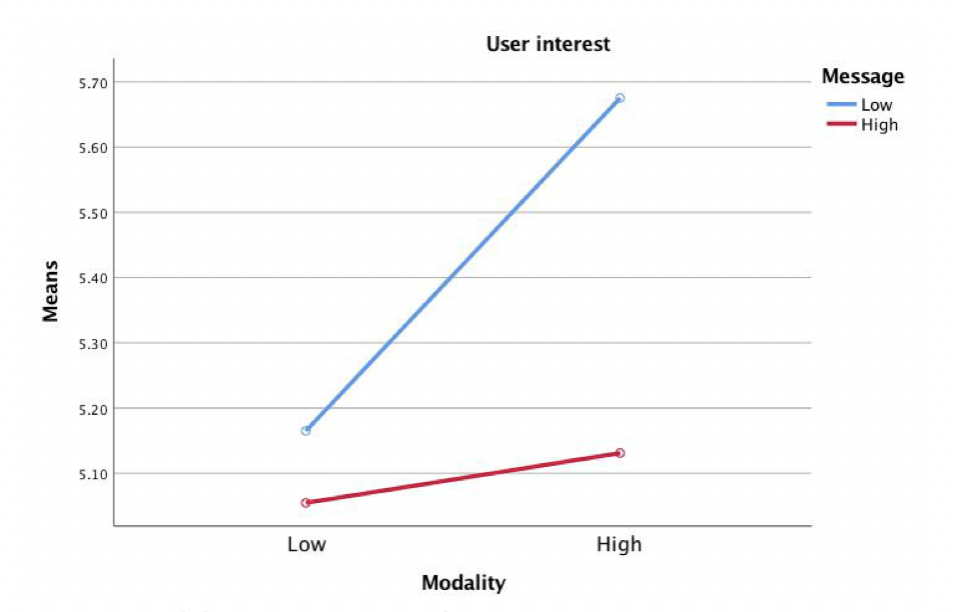
Modality interactivity and message interactivity effect on user interest.

According to the results of two 2-way analysis of covariance, there was no main effect of modality interactivity or message interactivity on user satisfaction; however, the interaction effect of modality interactivity and message interactivity on user satisfaction was significant (*F*_1,283_=4.52, *P=*.023, η^2^=.01). Participants in the high modality interactivity and high message interactivity condition (mean 4.70, SE 0.14) felt more satisfied compared to participants in the low modality interactivity condition and high message interactivity condition (mean 4.34, SE 0.14) (Figure 6). The main effect of message interactivity on user interest was also significant (*F*_1,283_=4.08, *P=*.044, η^2^=.01). Participants in the low message interactivity condition (mean 5.68, SE 0.16) were more interested in the health website user experience than participants in the high message interactivity condition (mean 5.06, SE 0.16) (Figure 7), answering the second research question.

## Discussion

### General

This study aimed to examine both the main effects and interaction effects of 2 types of interactivity presented on health websites regarding skin cancer. This study tries to illustrate the distinct effect of different types of interactivity in an empirical study and extends the literature by investigating skin cancer–related perceptions, interaction effects of different types of interactivity, and how interactivity impact user experience with health websites. This study has both theoretical and empirical implications.

### Behavioral Intentions

Different types of interactivity influenced behavioral intention to use sunscreen differently through different mediators. Message interactivity had a direct negative effect on the intention to use sunscreen. The reason could be that message interactivity takes more cognitive effort or the process of information acceptance was not favorable [6]. Therefore, individuals who only experienced message interactivity using the skin cancer website without additional modality interaction had lower intention to use sunscreen in the future. Modality interactivity had a direct positive effect on intention to use sunscreen. Individuals who experienced greater modality features on the website were more likely to use sunscreen in the future. Therefore, health researchers and health campaign designers can decrease message interactivity and increase modality interactivity on the health website to achieve the goal of promoting behavioral intention to use sunscreen.

### Attitudes and Knowledge

Attitudes and perceived knowledge were found to mediate the effects of different interactivity on intention to use sunscreen. Individuals who experienced a high level of modality features on the health websites tended to have more favorable attitudes toward the health websites, which includes their evaluation of website design, interaction with the features, and user experience of the whole website.

One contribution of this study is that both perceived knowledge and actual knowledge gain were tested to see if there was a difference between how these 2 types of knowledge were impacted by interactivity and how they influenced behavioral intention. Message interactivity had a positive effect on perceived knowledge. Participants in the high message interactivity condition felt more knowledgeable than participants in the low message interactivity condition. But there was no main effect of interactivity on actual knowledge gain (memory recall). Although participants perceived that their knowledge about skin cancer increased, their actual knowledge gain was not significantly affected by interactivity.

Skin cancer is the most preventable cancer compared to other forms of cancer and prevention of skin cancer has tremendous potential to save lives [35]. Preventive behaviors such as using sun protection are also relatively easy to perform for the general public. Lustria [21] found that the interactivity level of a website had a significant effect on the comprehension of the skin cancer content and attitudes toward health websites. This research extended the findings of previous research [21,24] testing interactivity on skin cancer–related outcomes and distinguished effects of different types of interactivity. Results of this study provide insights for the design of future skin cancer interventions, especially those using the web-based platforms [21,42,43].

These results also provide a direction for other health topics. Attitudes toward the website influenced behavioral intentions to perform actions related to the health topic, which should draw attention from health practitioners to website design. If the primary goal is to achieve health behavior change, health practitioners should focus on how to increase favorable attitudes toward health websites when designing health websites.

### User Experience

Previous digital health interventions have not focused on user experience with the media system or the program; however, the user experience may directly impact users’ absorption of the information and future behaviors [58]. Our study found support that interaction effect of modality interactivity and message interactivity had a positive influence on user satisfaction whereas participants experienced high modality interactivity and high message interactivity reported highest satisfaction scores on their interaction with the health websites. Additionally, participants in the low message interactivity condition scored the highest on the interest in their experience with the health websites. Future studies should investigate how user experience impact behavioral outcomes related to health.

### Theoretical Contribution and Implications in Health Interventions

In computer-mediated communication or human-computer interaction fields of study, scholars tend to use their own definitions or dimensions of interactivity in their studies [59]. Therefore, there is not a unified definition of interactivity. We used the MAIN model [6] as a theoretical background to conceptualize different types of interactivity and to map the relationships between interactivity, mediators, and behavioral outcomes. Previous studies have examined some interactivity models in the advertising field (eg, a dual-process model of interactivity effects [47]) or focused on building a moderation model of technological attributes (eg, mediated moderation model of interactivity [60]). However, there are not many studies explicitly assessing the influences of different interactivity in eHealth or mobile health (mHealth) interventions [59]. Employing interactivity in health interventions still requires additional empirical studies. Our findings lend support to most of the theoretically constructed hypotheses and demonstrates that different types of interactivity have different influences on health behavioral intention through different mediators. The technological affordances on the media systems need to be carefully defined and applied in eHealth and mHealth interventions.

This study also has empirical implications in designing health interventions or health campaigns. Previous studies have used interactivity as a general concept and have not differentiated among various technological functions, such as hyperlinks and 3D rotation function (eg, hyperlinks [61]; zoom-in and zoom-out, pan and rotate [20]; navigation tools and hyperlinks [21]). This study distinguished message interactivity and modality interactivity and operationalized these 2 types of interactivity with different technological features in an experimental study and explicitly examined the distinct effects of different types of interactivity on a health website, which has filled the previous literature gap in interactivity research and has added methodological implications to empirical eHealth research. Future health interventions using interactive websites or any interactive futures on the interface could apply the findings from this study to achieve desired goals.

### Limitations and Future Studies

Like many studies, this study has some limitations. First of all, Mechanical Turk data were used in this study. Mechanical Turk data provide more socioeconomically and demographically diverse samples than those obtained from college and traditional internet sampling [54]. However, as an online experiment, we had less control during the data collection process. The process of how viewers browsed the websites was not clear. Additionally, the lack of control could also influence participants’ experience with the website. While participants were directed to use the features of the website, we do not have data related to the length of time spent on each webpage or the number of interactivity features accessed. While only participants who passed the manipulation check remained in the study, there could also be differences based on the level of interaction with which participants engaged with the website, which we were unable to examine in this study. However, the use of an online experiment mimics the real situation of how people may view health websites at home or other places in their daily life instead of a research lab. This may lend additional external validity to the study. Given the rapid development of new technologies on websites, future studies should also employ new interactive features and establish user-centered websites with more professional functions.

Participants who had been diagnosed with skin cancer or had relatives who had skin cancer might be more engaged or be impacted more by skin cancer interventions. Future studies should distinguish the effects of different experiences with skin cancer. Additionally, the outcome variables in this study were attitudes and behavioral intentions instead of actual behaviors. People may overreport their behavioral intentions to perform healthy activities for social desirability. Therefore, future studies should aim at measuring actual behavior change such as sun protection behaviors to investigate the behaviors in real-world situations. A longitudinal study is needed to evaluate how interactive features work in changing people’s health behaviors, which might be the ultimate goal of health campaigns and interventions.

### Conclusions

This study, which used a 2×2 experimental design to assess 2 different types of interactivity, has contributions related to designing effective health websites for health interventions. Different types of interactivity along with attitudes, knowledge, and behavioral intention were examined to map the mediating relationships between independent and dependent variables. Both modality interactivity and message interactivity had direct positive effects on behavioral intention to use sunscreen. Modality interactivity also had an indirect positive effect on behavioral intention through attitudes toward the website. Message interactivity had an indirect effect on behavioral intention to use sunscreen via perceived knowledge. To design a health intervention or campaign in the digital age, health researchers and practitioners could employ interactive features in their designs accordingly. This study has important insights for health practitioners who have different aims when designing health websites for eHealth and mHealth interventions.

## Abbreviations

MAIN: Modality, Agency, Interactivity, and Navigability
mHealth: mobile health
